# Design of High-Reliability Micro Safety and Arming Devices for a Small Caliber Projectile

**DOI:** 10.3390/mi8080234

**Published:** 2017-07-28

**Authors:** Dakui Wang, Wenzhong Lou, Yue Feng, Xinzhao Zhang

**Affiliations:** National Key Laboratory of Electro-Mechanics Engineering and Control, School of Mechanical-Electronic Engineering, Beijing Institute of Technology, Beijing 100081, China; 3120140080@bit.edu.cn (D.W.); louwz@bit.edu.cn (W.L.); zhangxinzhao@bit.edu.cn (X.Z.)

**Keywords:** micro S & A device, centrifugal insurance mechanism, small caliber projectile platform, theoretical, simulation and experimental methods

## Abstract

With the development of micro technology, the fuse for small-caliber projectiles tends to be miniaturized and intelligent, the traditional fuse no longer meets the requirements. In this paper, we demonstrate a micro safety and arming (S & A) device with small volume and high reliability in small caliber projectile platforms. The working principle of S & A devices is that a centrifugal insurance mechanism could deform under a centrifugal load and thus cause fuse safety arming. The centrifugal insurance mechanism is designed theoretically, verified by simulation and experimental methods. The experimental results show that, when the rotary speed is over 36,000 rpm, the fuse was armed safely. In addition, the experimental, simulation, and theoretical results are basically consistent, and indicate that the centrifugal insurance mechanism meets the expected criteria.

## 1. Introduction

Fuse is an important part of the ammunition to achieve precision aim and efficient damaging ability [1,2,3]. Once certain conditions such as rotary speed have been reached, the safety and arming (S & A) device must arm reliably and the fuse enters the state of readiness [4,5,6]. Traditional S & A devices can accomplish the safety and arming functions of a fuse, however, this is not available for modern weapon because of its large size, low accuracy, and poor anti-overload capability [7,8,9,10]. The miniaturization of the S & A device for fuses contributes a lot to the system control of munitions, because weapons with S & A devices of a smaller size can provide more space for other devices [11,12]. 

The application of micro technology can deal with this problem efficiently, which should bring a significant influence to the development of fuse [13,14]. Most micro S & A devices are through the environmental forces to be arming [15,16,17]. The representative micro S & A devices are as follows. A series of micro S & A devices designed by the team of Charles H. Robinson, which achieve arming through the recoil force [18,19,20,21,22,23]. The micro S & A devices are safely armed through the recoil and centrifugal forces, which are designed by the France Company of NEXER, the Indian Head research institute, and the KAMAN Company of America [24,25,26,27,28]. In addition, most of the setback and centrifugal insurance mechanisms in micro S & A devices are made of elastic beam and mass block.

In this paper, we designed a high-reliability micro S & A device in the small caliber projectile platform based on the expected design criteria. The size of the centrifugal insurance mechanism is determined theoretically, and the centrifugal insurance mechanism is studied under different conditions by simulation. Finally, the experimental results are presented, and the corresponding theoretical and simulation models are verified quantitatively.

## 2. Design

**Figure 1:**
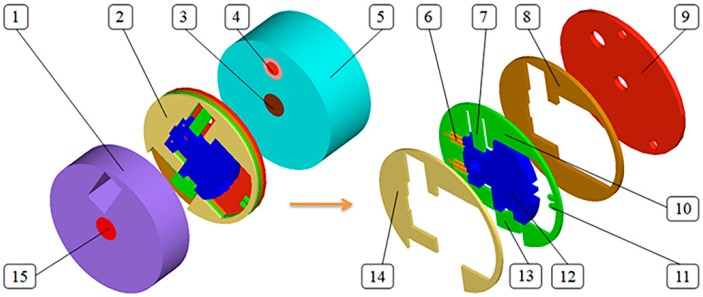
Designed Micro S & A device. **1**—Detonating tube base; **2**—Micro S & A module; **3**—Electric detonator; **4**—Electric thruster; **5**—Electric detonator base; **6**—Micro spring; **7**—Latching reed; **8**—Top plate; **9**—Cover plate; **10**—Middle plate; **11**—Latching mechanism; **12**—Explosion-proof slider; **13**—Centrifugal insurance mechanism; **14**—Bottom plate; **15**—Detonating tube.

We proposed a micro S & A device for use in small caliber projectile platforms, the concrete structure is as shown in Figure 1. Due to the limited size of small caliber projectile (The caliber is 35 mm), the size of the device is designed as Φ14 × 15 mm. Therein, the assembling process of the micro spring and the explosion-proof slider is that clipping two ends of the micro spring into the gaps of the explosion-proof slider and the middle plate, then both of them are fixed by the top and bottom plates. The working principle of the micro S & A device is as follows. Under the launching state of the small caliber projectile, micro S & A device withstands the setback and centrifugal force, the centrifugal insurance mechanism releases the explosion-proof slider. Because the launching state lasts a long time, the centrifugal insurance mechanism maintains the arming state. When ammunition flies a certain distance, the electric thruster works and deforms the latching read, the explosion-proof slider is released. Under the impact of the centrifugal force, the explosion-proof slider overcomes the tension of the micro spring and moves. Immediately afterward, the latching mechanism works and the fuse enters the state of readiness. 

The centrifugal insurance mechanism plays the most critical role in whether the micro S & A device can work properly or not. Therefore, it is necessary to conduct research on the centrifugal insurance mechanism.

According to the design criteria of the micro S & A device, the centrifugal insurance mechanism needs to meet the following conditions. First, the centrifugal insurance mechanism can reliably arm when the rotary speeds are in the range of 25,000 rpm and 40,000 rpm. Meanwhile, the centrifugal insurance mechanism is limited by the projectile shell outside the micro S & A device. Second, when the electric thruster works accidentally, under the ultimate rotary speed of 75,000 rpm, the centrifugal insurance mechanism could effectively lock the explosion-proof slider to ensure the safety of fuse.

In order to meet these conditions, we conducted the theoretical, simulation, and experimental studies of the centrifugal insurance mechanism.

## 3. Theoretical Analysis

**Figure 2:**
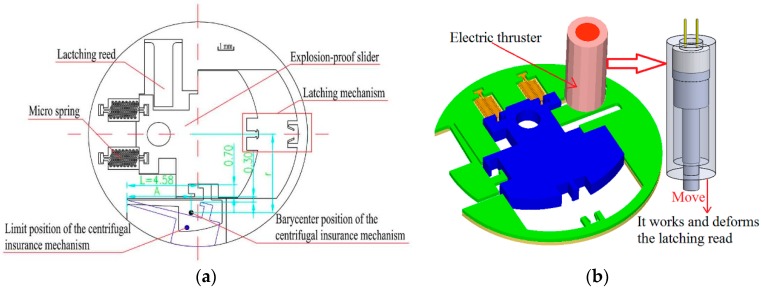
The design scheme of the micro safety and arming (S & A) module. (**a**) The 2D model of the micro S & A module; (**b**) The 3D model of the micro S & A module.

The micro S & A module is designed and its design scheme as shown in Figure 2, where the design parameters of centrifugal insurance mechanism are included. The size of the microelectromechanical systems (MEMS) S & A module is Φ12 × 0.3 mm.

In this paper, the materials of the micro spring and the other parts of the micro S & A module are nickel and beryllium bronze. The material parameters are shown in Table 1.

Assume that the centrifugal insurance mechanism is an elastic beam structure, the deflection of the centrifugal insurance mechanism is analyzed by the beam deflection curve equation in material mechanics. The deflection equation of the centrifugal insurance mechanism is calculated by Equation (1).

(1) $$W=-\frac{Fx^{2}}{6EI}\left(3A-x\right).$$

when x=L, the deflection value at the position of L is obtained.

(2) $$W=-\frac{Fx^{2}}{6EI}\left(3A-L\right).$$

Moreover, the centrifugal force F is equal to

(3) $$F=mA\omega^{2}=\rho vA\omega^{2}=\rho s_{0}hA\omega^{2}.$$

Wherein, ρ is the material density, $s_{0}$ is surface area and h is the thickness of the centrifugal insurance mechanism, A is the rotation radius of centroid in the centrifugal insurance mechanism, and ω is the rotary speed.

In addition, the moment of inertia for centrifugal insurance mechanism is expressed as follows.

(4) $$I_{z}=\;\frac{hb^{3}}{12}.$$

Taking the cross section of the root for the centrifugal insurance mechanism as the research object, the deflection W is achieved.

(5) $$W=-\frac{\rho s_{0}hr\omega^{2}L^{2}}{E\frac{hb^{2}}{2}}\left(3A-L\right)=-\frac{2\rho s_{0}r\omega^{2}L^{2}}{Eb^{3}}\left(3A-L\right).$$

Herein, the width of the beam section in the centrifugal insurance mechanism is b.

**Figure 3:**
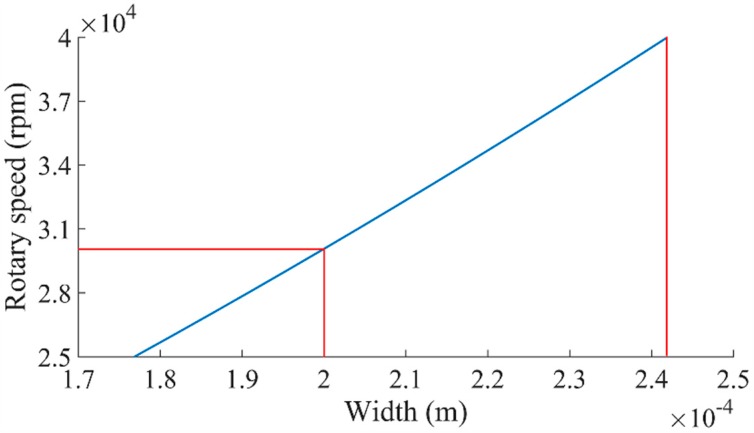
The theoretical calculation results of the centrifugal insurance mechanism.

According to the formula above, the formula does not contain the parameter of h. Therefore, the thickness has nothing to do with the motion of the centrifugal insurance mechanism. According to the design scheme as shown in Figure 2, we take $h=0.3\times 10^{-3}$ m, $L=4.88\times 10^{-3}$ m and $W=1\times 10^{-3}$ m. While the surface area ($s_{0}$), the barycenter position (A) and the rotation radius of the centrifugal insurance mechanism are determined by parameter b. According this, we take ω=25,000 rpm ~ 40,000 rpm and get the theoretical results as shown in Figure 3. As a result, it is found by Figure 3 that when $b=0.177\times 10^{-3}$–$0.242\times 10^{-3}$ m, the structure meets the design criteria first. We conduct the research by taking $b=0.2\times 10^{-3}$ m here and get the theoretical results as shown in Figure 3 when the rotary speeds are between 25,000 rpm and 40,000 rpm. Figure 3 shows that, when ω=30,100 rpm, the deflection of the centrifugal insurance mechanism could meet $W=1\times 10^{-3}$ m.

Moreover, when $b=0.2\times 10^{-3}$ m, we could obtain the mass and the rotation radius for centroid of the centrifugal insurance mechanism which are $m=1.714\times 10^{-5}$ Kg and $A=4.18\times 10^{-3}$ m, respectively. The structure strength of the centrifugal insurance mechanism is analyzed, and the expression of the bending stress of the beam is obtained.

(6) $$\sigma_{s}=\frac{6mA^{2}\omega^{2}}{hb^{2}}=1.2\;\mathrm{Gpa}.$$

Therefore, taking b, h, m, and A into Equation (6), we get the result as below.

(7) ω=27,047 rpm.

When the rotary speed reached the limiting value of 27,047 rpm, the plastic deformation appeared in the centrifugal insurance mechanism and the beam element was acted under the limiting rotary speed and plastic deformation occurred at the same time.

## 4. Simulation Analysis

### 4.1. Performance Simulation

**Figure 4:**
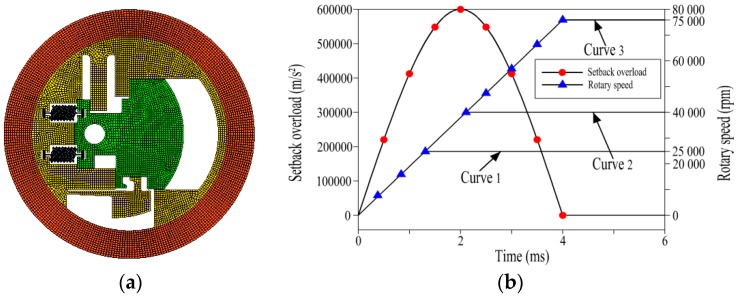
Simulation analysis of the micro S & A module in ANSYS/LS-DYNA. (**a**) The finite element model of micro S & A module; (**b**) The centrifugal and overload force application curve [4,16].

According to the theoretical analysis results above, the finite element model of the micro S & A module is built as shown in Figure 4a. The finite element method (FEM) is simulated by ANSYS/LS-DYNA (Version 17.0, ANSYS, Pittsburgh, PA, USA) explicit dynamic analysis software, wherein the element type of the model is MAT_PLASTIC_KINEMATIC (Version 17.0, ANSYS, Pittsburgh, PA, USA). Furthermore, the outermost loop is an additional model of the FEM, which is used to simulate the limit of the projectile shell to the centrifugal insurance mechanism.

According to the design criteria above, the following performance simulations are conducted.

**Figure 5:**
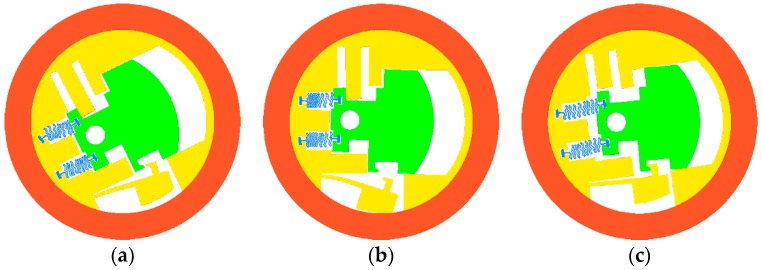
The simulation results for the micro S & A module under the different rotary speed curves. (**a**) curve 1 and the electric thruster does not work; (**b**) curve 2 and the electric thruster does not work; (**c**) curve 3 and the electric thruster works.

On the condition that the electric thruster does not work, the micro S & A module is simulated by loading the rotary speed curve 1 and curve 2, respectively, as shown in Figure 4b. When the simulation is performed under the rotary speed curve 1, the simulation result of Figure 5a is obtained. Figure 5a shows that the centrifugal insurance mechanism does not completely remove the constraints on the explosion-proof slider. When the simulation is carried out under the rotary speed curve 2, the simulation result (Figure 5b) indicates that the centrifugal insurance mechanism completely removes the constraints on the explosion-proof slider. Thus, the centrifugal insurance mechanism meets the design criteria first under the condition of simulation.

In the case that the electric thruster works, the simulation of the micro S & A module is performed under the rotary speed curve 3, as shown in Figure 4b. The result in Figure 5c illustrates that at the beginning of the projectile rotation, the explosion-proof slider moves and is stuck by the centrifugal insurance mechanism. When the rotary speed reaches its maximum value, because of the explosion-proof slider and the centrifugal insurance mechanism interlock, the explosion-proof slider cannot move, ensuring the safety of the fuse. Thus, the centrifugal insurance mechanism meets the second design criteria according to the simulation result.

### 4.2. Multi-Physics Coupling Simulation

Because the micro S & A module of the small caliber projectile is widely used in different platforms and regions, it is necessary to study the micro S & A module in different conditions. The centrifugal insurance mechanism of the micro S & A module is parametrically studied, considering its overall characteristics and weakness in the multi physical field.

**Figure 6:**
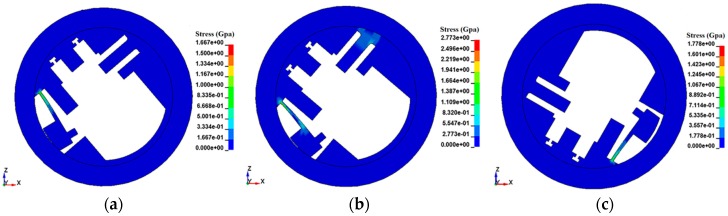
The simulation results of the micro S & A module under the different loads and temperatures. (**a**) Loading the centrifugal force (25 °C); (**b**) Loading the centrifugal force and setback overload (25 °C); (**c**) Loading the centrifugal force (−40 °C).

Nonlinear dynamic mechanics simulation is built using ANSYS/LS-DYNA under the condition of loading, the rotary speed curve 3 and the setback overload (shown in Figure 4b) at the temperatures of 25, 50, and −40 °C, respectively. The simulation results are concluded in Figure 6 and Table 2.

Table 2 tells us that the initial motion time of the centrifugal insurance mechanism is 1.57 ms under the centrifugal force, and the initial motion time of centrifugal insurance mechanism under the centrifugal force and setback overload is 1.56 ms, the two results are very close. The arming time of centrifugal insurance mechanism under the centrifugal force is 2.02 ms, which is over 0.04 ms than that under the centrifugal force and setback overload, the difference is very small. Meanwhile, Figure 6 indicates that the stress of centrifugal insurance mechanism under the centrifugal force and setback overload is greater than that under the centrifugal force. In conclusion, the setback overload can obviously increase the stress value of the structure, but has little effect on the arming time of the centrifugal insurance mechanism. 

The simulation results at the different temperatures could be concluded that the centrifugal insurance mechanism does not effectively achieve arming at low temperature (−40 °C). In addition, the centrifugal insurance mechanism could realize arming at a little higher temperature (50 °C) than the room temperature. The reason is mainly that the influence of low temperature on the material properties is much higher than that of high temperature.

**Figure 7:**
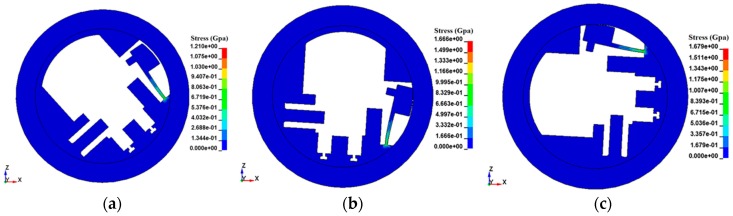
The simulation results of the micro S & A module under different rotary speeds. (**a**) 31,000 rpm; (**b**) 35,000 rpm; (**c**) 45,000 rpm.

**Figure 8:**
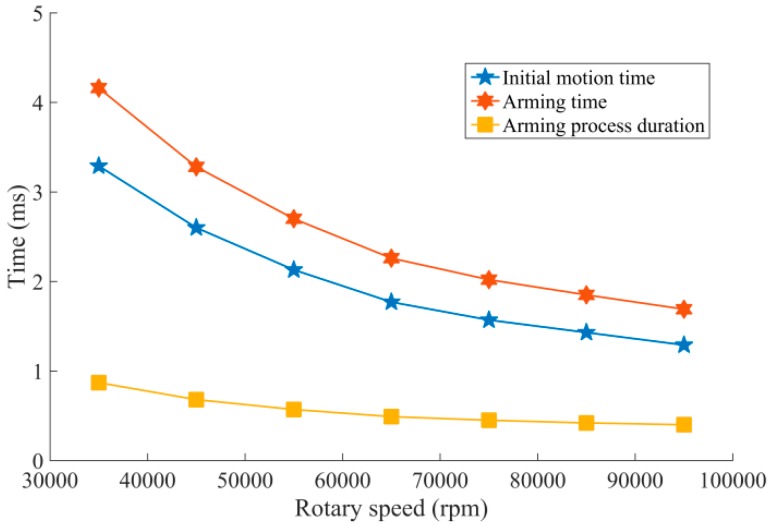
The fitting curves of arming-related time from simulation results.

Furthermore, we take the rotary speed 25,000 rpm as the initial value and 1000 rpm as a step length to explore the ultimate rotary speed of arming for the centrifugal insurance mechanism, and the simulation results are shown in Figure 7 and Figure 8. 

Figure 7 demonstrates that when the rotary speed reaches 31,000 rpm, plastic deformation appeared in the structure, and the centrifugal insurance mechanism arms when the rotary speed increases to 35,000 rpm. On the other hand, Figure 8 suggests that, with the increase of rotary speed, the initial motion time, the arming time, and the arming process duration of the centrifugal insurance mechanism all tend to be decreased. The reason is when the centrifugal insurance mechanism owns the same deformation, the faster rotary speed, the shorter deformation period.

## 5. Experimental Analysis

**Figure 9:**
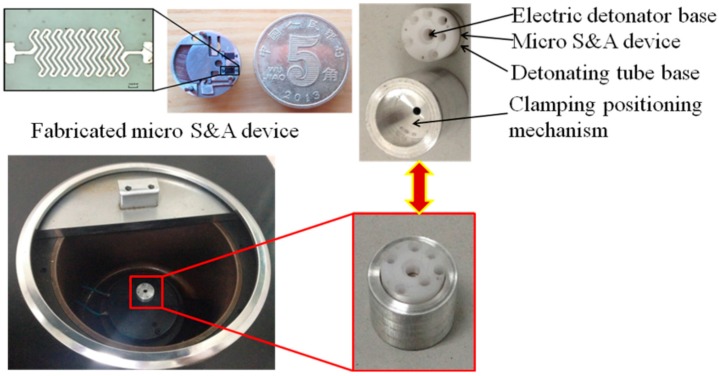
The fabricated micro S & A module and the experimental setups.

The micro spring, based on the nickel material and ultraviolet-LIGA (German Acronym for Lithographie, Galvanformung, Abformung) process, is fabricated. Beryllium bronze material and low-speed wire-cutting electrical discharge processing technology are used to manufacture the other parts of micro S & A module. Figure 9 shows the fabricated micro S & A module.

Combined with the simulation project and results, two groups of experiments should be performed. Firstly, under the condition of the electric thruster malfunction, we take the rotary speed 25,000 rpm as the initial value and 1000 rpm as a step length to explore the ultimate rotary speed of arming for the centrifugal insurance mechanism. Then, when the electric thruster works malfunctions, the experiments are conducted under 50,000 rpm (this is the maximum speed of the centrifuge) of the rotary speed. The experimental equipment is shown in Figure 9. According to whether the electric thruster works or not, four group experiments of each situation are carried out, respectively.

**Figure 10:**
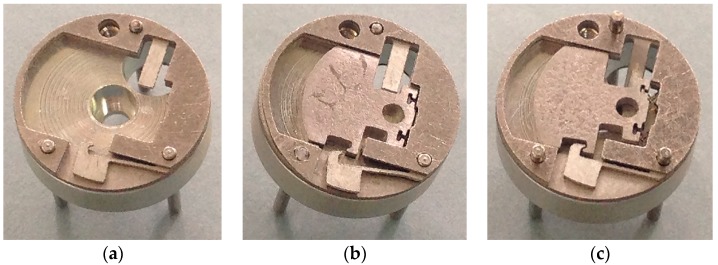
The states of arming mechanism under the different conditions. (**a**) 34,000 rpm and the electric thruster does not work; (**b**) 36,000 rpm and the electric thruster does not work; (**c**) 50,000 rpm and the electric thruster works.

On the condition that the electric thruster does not work, the ultimate rotary speed of the micro S & A module arming is seen in Table 3. The experimental results of the micro S & A device under the rotary speeds of 34,000 rpm and 36,000 rpm are obtained in Figure 10a,b, respectively. In the case that the electric thruster works, the experimental result of the micro S & A device under the rotary speeds of 50,000 rpm as shown in Figure 10c.

Figure 10a shows that plastic deformation occurs in the centrifugal insurance mechanism, but the structure does not achieve arming. Figure 10b indicates that the centrifugal insurance mechanism completely achieves arming. Figure 10c demonstrates that the centrifugal insurance mechanism effectively locks the explosion-proof slider. By contrast with the simulation results as shown in Figure 5, the correctness of the models is verified.

Table 3 tells us that the minimum rotary speed of arming for the centrifugal insurance mechanism and the average value of the rotary speeds is 36,000 rpm. By comparing with the simulation and theoretical result of the ultimate rotary speed, it indicates that the experimental result is slightly larger than the simulation result and theoretical result, the reason is mainly that the friction and damping are ignored in the simulation and theoretical analysis. The experimental result, simulation result, and theoretical result are basically consistent. It can be proved that the designed centrifugal insurance mechanism can meet the design requirements.

## 6. Conclusions

In this paper, we designed and micro fabricated a micro S & A device for use in small caliber projectile platforms, which is designed theoretically, and verified by simulation and experimental methods. When the rotary speed is over 36,000 rpm, the fuse was safely armed. Through the multi-physics coupling simulation results, we proved that the designed micro S & A device is quite suitable for working in the temperature equaled to or slightly higher than 25 °C. It indicates that the centrifugal insurance mechanism meets the design criteria we defined, and such a design method is quite effective and useful for the small caliber projectile.

## Figures and Tables

**Table 1:** The material parameters of the beryllium bronze and nickel [17].

Material	Density (kg/m^3^)	Young’s Modulus (GPa)	Poisson’s Ratio	Yield Strength (GPa)
Beryllium bronze	8.3 × 10^3^	133	0.33	1.2
Nickel	8.9 × 10^3^	150	0.31	0.815

**Table 2:** Simulation results under different loads and temperatures.

Load	Initial Motion Time (ms)	Arming Time (ms)	Arming Process Duration (ms)
Centrifugal force (25 °C)	1.57	2.02	0.45
Centrifugal and setback force (25 °C)	1.56	1.98	0.42
Centrifugal force (50 °C)	1.58	2.01	0.43
Centrifugal force (−40 °C)	Arming failed	Arming failed	Arming failed

**Table 3:** The ultimate rotary speed of the micro S & A module arming.

Group Number	1	2	3	4
Rotary speed (rpm)	36,000	37,000	35,000	36,000
Average (rpm)	36,000			
